# Manipulation of Lipid Metabolism During Normothermic Machine Perfusion: Effect of Defatting Therapies on Donor Liver Functional Recovery

**DOI:** 10.1002/lt.25439

**Published:** 2019-06-20

**Authors:** Yuri L. Boteon, Joseph Attard, Amanda P. C. S. Boteon, Lorraine Wallace, Gary Reynolds, Stefan Hubscher, Darius F. Mirza, Hynek Mergental, Ricky H. Bhogal, Simon C. Afford

**Affiliations:** ^1^ Liver Unit Queen Elizabeth Hospital, University Hospitals Birmingham National Health Service Foundation Trust Birmingham United Kingdom; ^2^ Department of Cellular Pathology Queen Elizabeth Hospital, University Hospitals Birmingham National Health Service Foundation Trust Birmingham United Kingdom; ^3^ National Institute for Health Research Birmingham Biomedical Research Centre University Hospitals Birmingham National Health Service Foundation Trust Birmingham United Kingdom; ^4^ Centre for Liver and Gastrointestinal Research Institute of Immunology and Immunotherapy, College of Medical and Dental Sciences, University of Birmingham Birmingham United Kingdom; ^5^ The Royal Marsden, Department of Academic Surgery Fulham Road Chelsea London

## Abstract

Strategies to increase the use of steatotic donor livers are required to tackle the mortality on the transplant waiting list. We aimed to test the efficacy of pharmacological enhancement of the lipid metabolism of human livers during ex situ normothermic machine perfusion to promote defatting and improve the functional recovery of the organs. Because of steatosis, 10 livers were discarded and were allocated either to a defatting group that had the perfusate supplemented with a combination of drugs to enhance lipid metabolism or to a control group that received perfusion fluid with vehicle only. Steatosis was assessed using tissue homogenate and histological analyses. Markers for lipid oxidation and solubilization, oxidative injury, inflammation, and biliary function were evaluated by enzyme‐linked immunosorbent assay, immunohistochemistry, and in‐gel protein detection. Treatment reduced tissue triglycerides by 38% and macrovesicular steatosis by 40% over 6 hours. This effect was driven by increased solubility of the triglycerides (*P* = 0.04), and mitochondrial oxidation as assessed by increased ketogenesis (*P* = 0.008) and adenosine triphosphate synthesis (*P* = 0.01) were associated with increased levels of the enzymes acyl‐coenzyme A oxidase 1, carnitine palmitoyltransferase 1A, and acetyl‐coenzyme A synthetase. Concomitantly, defatted livers exhibited enhanced metabolic functional parameters such as urea production (*P* = 0.03), lower vascular resistance, lower release of alanine aminotransferase (*P* = 0.049), and higher bile production (*P* = 0.008) with a higher bile pH (*P* = 0.03). The treatment down‐regulated the expression of markers for oxidative injury as well as activation of immune cells (CD14; CD11b) and reduced the release of inflammatory cytokines in the perfusate (tumor necrosis factor α; interleukin 1β). In conclusion, pharmacological enhancement of intracellular lipid metabolism during normothermic machine perfusion decreased the lipid content of human livers within 6 hours. It also improved the intracellular metabolic support to the organs, leading to successful functional recovery and decreased expression of markers of reperfusion injury.

**Abbreviations**
4‐HNE: 4‐hydroxynonenal
8‐HOdG: 8‐hydroxy‐2‐deoxyguanosine
ACOX1: acyl‐coenzyme A oxidase 1
ALT: alanine aminotransferase
AST: aspartate aminotransferase
ATP: adenosine triphosphate
AUC: area under the curve
cAMP: cyclic adenosine monophosphate
CAR: constitutive androstane receptor
CIT: cold ischemia time
CoA: coenzyme A
CPT1A: carnitine palmitoyltransferase form 1A
DBD: donation after brain death
DCD: donation after circulatory death
DMSO: dimethyl sulfoxide
ECD: extended criteria donor
ET: Eurotransplant
FA: fatty acid
GGT: gamma‐glutamyl transpeptidase
H & E: hematoxylin‐eosin
HA: hepatic artery
HDL: high‐density lipoprotein
IRI: ischemia/reperfusion injury
IL: interleukin
IQR: interquartile range
IRS: modified immunoreactive score
LD: lipid droplet
MaS: macrovesicular steatosis
MiS: microvesicular steatosis
NHSBT: National Health Service Blood and Transplant
NMP: normothermic machine perfusion
PAS: periodic acid–Schiff
PCO_2_: partial pressure of carbon dioxide
PPAR: peroxisome proliferator‐activated receptor
P‐TG: perfusate triglyceride
PXR: pregnane X receptor
PV: portal vein
ROS: reactive oxygen species
SCS: static cold storage
TG: triglyceride
TKB: total ketone bodies
TNF‐α: tumor necrosis factor α
T‐TG: tissue triglyceride
VLDL: very low density lipoprotein
WIT: warm ischemia time

Steatosis is caused by the abnormal metabolism of fatty acids (FAs) in hepatocytes, resulting in intracytoplasmic accumulation of triacylglycerol as lipid droplets (LDs).^1^ In the context of organ donation, livers with large intracytoplasmic LDs displacing the cell nucleus, ie, macrovesicular steatosis (MaS), are more vulnerable to ischemia/reperfusion injury (IRI) during standard static cold storage (SCS). This is predominantly due to impaired mitochondrial function, poor microcirculation, and exaggerated inflammatory response leading to tissue damage.^2^ The exacerbated IRI is then associated with impaired early functional recovery and a high risk of early allograft dysfunction.^2^, ^3^, ^4^ Therefore, steatosis is one of the main reasons worldwide that transplant teams decline donor livers.^2^, ^5^

Animal models have shown that normothermic machine perfusion (NMP) of the liver alone is able to improve intracellular lipid metabolism and promote steatosis reversal or defatting.^6^, ^7^ However, a recent study involving NMP of steatotic human livers for 24 hours did not show a decrease in tissue steatosis.^5^ In a murine model, a combination of drugs (peroxisome proliferator‐activated receptor [PPAR] α ligand GW7647, PPARδ ligand GW501516, pregnane X receptor ligand hypericin, the constitutive androstane receptor ligand scorparone, the cyclic adenosine monophosphate [cAMP] activator forskolin, and the insulin‐mimetic adipokine visfatin) administered during NMP enhanced the hepatic lipid metabolism and reduced tissue triglycerides (T‐TGs) by 50% over 3 hours of perfusion.^6^ Similar results were observed with the glial cell line–derived neurotrophic factor.^8^ However, whether those observations could be reproduced in human livers is still to be determined. In addition, the organs in these studies were not exposed to ischemic injury, as is the case in liver transplantation. Therefore, the defatting process in the context of liver transplantation remains to be determined.

We aimed to study the feasibility of the delivery of this combination of drugs to human donor livers undergoing end‐ischemic NMP within the regular process of organ donation and cold preservation. The impact of this intervention on the mobilization and metabolization of intracellular lipids leading to defatting was assessed, together with its effects on recovery of the metabolic activity of the organs.

## Patients and Methods

### Study Design and Source of Discarded Donor Livers

Ten human livers discarded for transplantation were randomly allocated to the experimental groups using the covariate adaptive randomization method that accounted for donor type and cold ischemia time (CIT). These were subsequently submitted to NMP for 12 hours after variable periods of SCS. The criterion for inclusion of an organ in the study was a rejection for transplantation due to the macroscopic assessment of steatosis by the transplant and/or retrieval surgeon. If a donor biopsy was taken beforehand, the histological grade of steatosis was recorded. However, absence of histological assessment of steatosis was not an exclusion criterion in the study because donor liver biopsy is not routinely performed in the United Kingdom, and even when a biopsy was done, this information was not always available at the time of the inclusion of the organ in the study. The defatting group had the perfusion fluid supplemented with a combination of drugs to improve lipid metabolism (defatting cocktail; 5 livers), and a control group (5 livers) received vehicle only in the perfusion fluid (dimethyl sulfoxide [DMSO] <0.01%). The comprehensive protocols for sampling (including core needle liver biopsy and perfusate and bile analysis) and data collection are presented in the Supporting Materials.

All study livers were originally retrieved with the intention of transplantation as per policy of the National Health Service Blood and Transplant (NHSBT). The organs were then rejected for transplantation by all UK centers and offered for research by the NHSBT. The authors had no influence in the process of declining donor organs, which was done by the transplant surgeon on call in the different transplant centers. The authorization for research use of the organ is obtained by the specialist nurse in organ donation in accordance with NHSBT guidelines. Ethical approval for the study was obtained from the London‐Surrey Borders National Research Ethics Service (reference 13/LO/1928) and the NHSBT ethics committee (reference 06/Q702/61).

### Liver Perfusion Procedure

After arrival at our center, the organs were prepared following the standard bench preparation as described elsewhere.^9^ The cystic duct was ligated, and a 10‐Fr bile cannula was inserted in the common bile duct. Liver Assist (Organ Assist, Groningen, the Netherlands) was the device used for the perfusions (details are provided in the Supporting Methods). The perfusate consisted of 3 units of packed red blood cells, 5% wt/vol human albumin solution, and additional drugs as specified in Supporting Table 1.

### The Defatting Cocktail of Drugs

A previously published cocktail of drugs^6^ (10 μM of forskolin; 1 μM of GW7647; 10 μM of hypericin; 10 μM of scoparone; 0.4 ng/mL of visfatin; 1 μM of GW501516) was supplemented with 0.8 mM of L‐carnitine diluted in DMSO and added in the perfusate for the defatting group when the perfusate reached 37°C. The control group received an equal amount of DMSO (<0.01% vol/vol) in the perfusate within the same time frame. The absence of in vitro cytotoxicity of the combination of drugs on primary human liver cells has been previously published by our group.^10^ Moreover, detailed pharmacodynamics of the defatting agents was recently reviewed.^11^ Drugs were obtained from Sigma‐Aldrich (St. Louis, MO), and a list is provided in the Supporting Methods.

### Histological Evaluation

Tissue biopsies were embedded in paraffin and cut in sections of 4 μm. Staining with hematoxylin‐eosin (H & E) was performed to assess necrosis, grade of steatosis, preexisting acute or chronic liver injury, and periodic acid–Schiff (PAS) for glycogen stores. MaS was defined as the presence of a large LD filling up the hepatocytes and displacing the nucleus to the periphery. Microvesicular steatosis (MiS) refers to the presence of numerous small LDs in the hepatocytes (“foamy” aspect) without affecting cell nuclei position. MaS was graded based on the percentage of hepatocytes involved (none, <5%; mild, 5%‐30%; moderate, >30%‐60%; and severe, >60%). MiS was reported as present or absent. Histological assessment was done under lower magnification (magnification ×4 to ×10) and confirmed under higher magnification (magnification ×20 to ×40), if required. It was conducted by 2 independent pathologists (G.R. and S.H.) blinded to the study group.

### Assessment of Lipid Metabolism

Perfusate total cholesterol, high‐density lipoprotein (HDL), and perfusate triglycerides (P‐TGs) were measured at the hospital clinical laboratories. T‐TG was assessed from tissue homogenates using a commercially available kit (number 10010303; Cayman Chemical, Miami, FL) following the manufacturer’s guidelines. Percentages were used to compare the differences in T‐TG between 2 time points (the difference between levels at 6 or 12 hours and time 0, divided by the time 0 value, multiplied by 100). Total ketone bodies (3‐hydroxybutyric acid and acetoacetic acid) were measured in the perfusate using a commercial kit (MAK134; Sigma‐Aldrich) following the manufacturer’s instructions.

In‐gel fluorescent protein staining was performed to investigate changes across groups in the transcription of 3 key enzymes in the intracellular lipid metabolism:

1. Acetyl‐coenzyme A (CoA) synthetase promotes the reaction of CoA with FAs generating the substrate required for mitochondrial β‐oxidation of FAs.
2. The liver‐specific 1A form of carnitine palmitoyltransferase form 1A (CPT1A) responsible for the shuttle of acetyl‐CoA into the mitochondrial matrix for oxidation.
3. Peroxisomal acyl‐coenzyme A oxidase 1 (ACOX1), an essential enzyme for the peroxisomal oxidation of medium‐ to long‐chain FAs.

Detailed techniques and the list of antibodies are provided in the Supporting Materials.

### Assessment of the Liver Metabolism

Parameters used in our unit as indicators of ongoing liver metabolism were applied to define whether organs would be potentially transplantable.^12^ Other parameters, such as the dynamics of lactate clearance, bile quality (assessed by bile pH), urea production, release of transaminases in the perfusate, and oxygen uptake, were also investigated. For analysis of dynamic changes in lactate metabolism over time, the area under the curve (AUC) of lactate concentrations in the perfusate between 0 and 12 hours was analyzed. Oxygen uptake was calculated as the difference between the oxygen inflow and the outflow in kPa.

### Biochemistry Analysis

Markers of hepatocellular injury (alanine aminotransferase [ALT] and aspartate aminotransferase [AST]), biliary injury/function (gamma‐glutamyl transpeptidase [GGT]), and protein release (albumin and total protein) were measured in the perfusate (time points in the sampling protocol).

### Cellular Energy Status Assessment

Levels of adenosine triphosphate (ATP) were assessed using a fluorometric commercial kit from liver tissue homogenates as per the manufacturer’s recommendations (MAK190; Sigma‐Aldrich). Levels were normalized to mg of protein.

### Assessment of Oxidative Injury and Inflammation

Immunohistochemistry was performed on formalin‐fixed, paraffin‐embedded sections for the expression of the marker of cell membrane phospholipid peroxidation 4‐hydroxynonenal (4‐HNE). To assess tissue inflammation, CD11b, an integrin on the surface of activated leukocytes, and CD14, a lipopolysaccharide receptor that is part of the toll‐like receptor 4 signalosome and participates in the development of the IRI, were investigated. For the detailed technique and antibodies list, refer to Supporting Methods. Staining expression was scored using a semiquantitative scoring system, the modified immunoreactive score (IRS).^13^ This takes into consideration intensity of staining (0, no color reaction; 1, mild reaction; 2, moderate reaction; and 3, intense reaction) and its distribution (0, no positive cells; 1, <10% positive cells; 2, 10%‐50% positive cells; 3, 51%‐80% positive cells; and 4, >80% positive cells). A final score between 0 and 12 is obtained by multiplying the 2 parameters.

Perfusate tumor necrosis factor α (TNF‐α), interleukin (IL) 10, and IL1β levels were determined using sandwich enzyme‐linked immunosorbent assay (RAB0476, RAB0244, RAB0273; Sigma‐Aldrich); 8‐hydroxy‐2‐deoxyguanosine (8‐HOdG) was assessed in the perfusate using a commercially available kit (ab201734; Abcam, Cambridge, MA) following the manufacturer’s instructions. All samples were tested in duplicate.

### Statistical Analysis

Continuous variables were expressed as median with interquartile range (IQR) and categorical variables as an absolute number with percentage frequencies. Comparisons between groups were performed using 2‐tailed Fisher’s exact test for categorical variables, Mann‐Whitney U test or Student *t* test for independent continuous variables, and Wilcoxon signed‐rank test for repeated measurements over time on the same sample. Correlations between variables were analyzed using Pearson’s correlation coefficient. The AUC was calculated using the trapezoidal rule. The statistical level of significance was set at *P* < 0.05. GraphPad Prism, version 6.04 (GraphPad Software, La Jolla, CA) software was used for all statistical analyses and graph creation.

## Results

### Donor Demographics and Perfusion Parameters

Overall median donor age was 51 years (IQR, 47‐58 years), median donor risk index was 2.0 (2.0‐2.1), and median CIT was 737 minutes (717‐805 minutes). Each study group included 3 donation after brain death (DBD) and 2 donation after circulatory death (DCD) livers. The groups were comparable in terms of donor characteristics and preservation times. Details are provided in Table 1 and Supporting Table 2.

**Table 1:** Donor Demographics, Liver Characteristics, and Machine Perfusion Data

Characteristic	Defatting (n = 5)	Control (n = 5)	*P* Value
Donor information			
Age, years	52 (47‐61)	49 (44‐66)	0.98
DCD livers	2 (40%)	2 (40%)	>0.99
Sex, male	3 (60%)	3 (60%)	>0.99
Height, cm	174 (162‐184)	172 (167‐184)	0.72
Body weight, kg	86 (75‐100)	95 (75‐105)	0.67
Body mass index, kg/m^2^	30 (28‐34)	28 (25‐34)	0.52
Donor risk index	2.0 (1.9‐2.8)	2.0 (1.8‐2.1)	0.22
UK donor liver index	1.1 (1.0‐1.8)	1.3 (0.9‐1.8)	0.94
ET donor risk index	1.9 (1.8‐3.1)	2.0 (2.0‐2.5)	0.75
Peak ALT, IU/L	66 (39‐99)	104 (39‐109)	0.38
Peak GGT, IU/L	110 (46‐239)	355 (31‐369)	0.29
Days on ventilator	2 (2‐4)	3 (1‐4)	0.64
Liver characteristics			
Liver weight, g	1874 (1731‐2362)	2130 (1775‐2228)	0.93
Donor WIT, minutes	12 (10‐14)	13 (13‐13)	0.70
DCD CIT, minutes	774 (729‐820)	770 (740‐800)	0.65
DBD CIT, minutes	754 (727‐788)	723 (621‐729)	0.29
Machine perfusion parameters			
Lactate, mmol/L			
Start (0 hours)	11.1 (9.2‐14.4)	10.6 (9.4‐11.8)	>0.99
Highest	11.6 (10.4‐16.3)	14.7 (12.1‐18.8)	0.38
Lowest	0.9 (0.3‐1.8)	1.6 (0.7‐11.7)	0.14
Last (12 hours)	1.9 (0.6‐2.7)	2.2 (1.0‐16.0)	**0.003**
Sodium bicarbonate 8.4% supplementation, mL	20 (15‐27)	40 (30‐90)	**0.04**
Total bile production, mL/hour	1.7 (1.6‐2.6)	0.6 (0.2‐1.6)	**0.02**
Bile pH (12 hours)	7.8 (7.7‐8.0)	7.3 (7.1‐7.6)	**0.02**
Median arterial flow, mL/minute	360 (283‐405)	286 (167‐343)	0.09
Median PV flow, mL/minute	1316 (1232‐1515)	1020 (900‐1302)	0.06
Viability achievement	5 (100%)	2 (40%)	**0.04**

NOTE:

Data are given as mean (IQR). Bolded *P* values are statistically significant.

**Figure 1:**
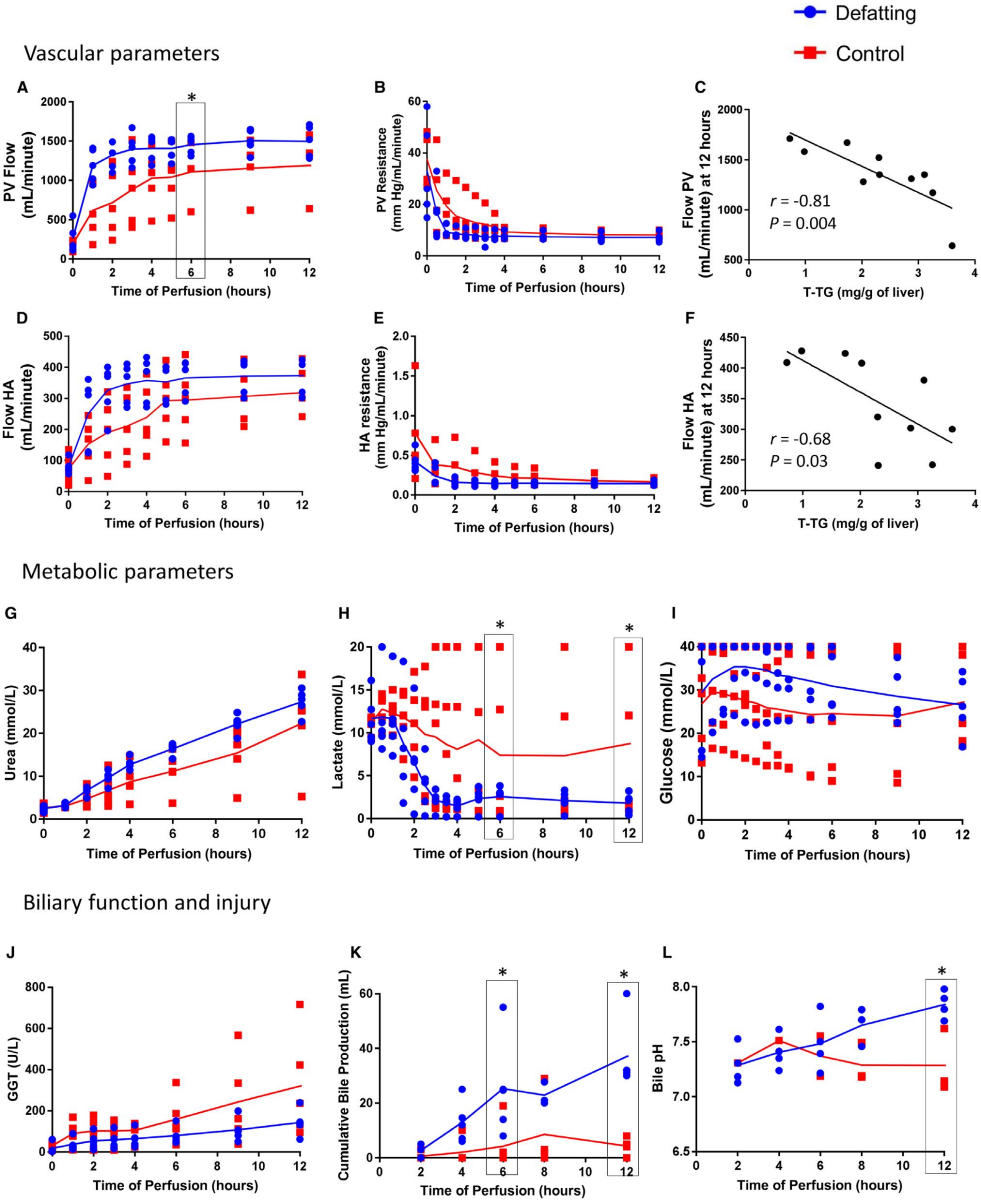
Perfusion parameters. (A‐F) The vascular parameters of the livers during perfusion. (A) The PV flow rate was higher in the defatted livers throughout the perfusion in comparison with the control group, (B) which was associated with a faster decrease in vascular resistance. There was a strong negative correlation between T‐TG levels and vascular flows at 12 hours on the (C) PV and (F) HA. (D) The HA flow rate with trends toward higher values for the defatting group, (E) which is potentially associated with the faster decrease in HA vascular resistance at the beginning of perfusion. (G‐I) Metabolic parameters of the livers during machine perfusion. (G) Perfusate urea concentration increased significantly more in the defatting group in comparison with the control group, which also had a rising trend. (H) Defatted livers metabolized perfusate lactate more effectively than the control group, reaching lower median concentrations. (I) Glucose levels peaked during the initial 2 hours of perfusion and then decreased steadily. In the control group, figures were stable initially and then tended to increase toward the end of the perfusion period. (J‐L) Data from biliary function and injury. (J) GGT levels in the perfusate increased significantly more in the control group. Concomitantly, treated livers produced significantly (K) more bile throughout the perfusion (L) with a higher bile pH. In all panels, the dots represent individual organs at the time points listed and the line indicates the median of the values for each group. Comparisons between groups were made with the Mann‐Whitney U test. *r* = Pearson’s correlation coefficient. *Statistical significance at *P* < 0.05.

Treated livers developed higher median portal vein (PV) flows (*P* = 0.06) with lower vascular resistance (*P* = 0.005) from the start until 12 hours of perfusion. There was a trend of higher median hepatic artery (HA) flows in the defatting group (*P* = 0.09) with a significantly lower HA resistance (*P* = 0.003) during this period. There was a strong correlation between T‐TG and flows at times 6 and 12 hours on the HA (*r* = –0.66, *P* = 0.04 and *r* = –0.68, *P* = 0.03, respectively) and PV (*r* = –0.79, *P* = 0.007 and *r* = –0.81, *P* = 0.004, respectively). Vascular parameters of the livers during perfusion are shown in Fig. 1.

### Liver Parenchyma Histology

**Figure 2:**
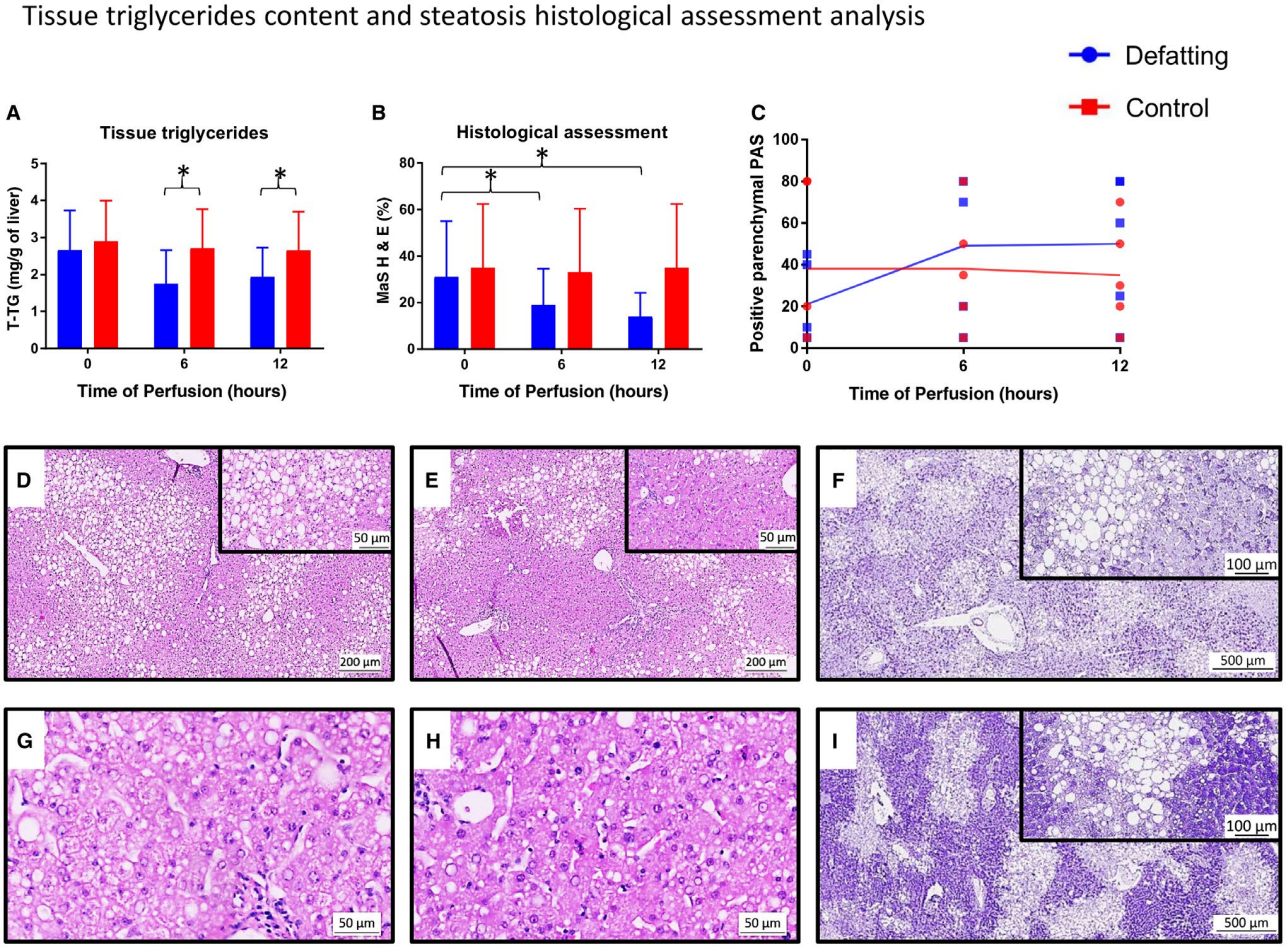
T‐TGs content and histological steatosis assessment analysis. (A) Homogenate T‐TGs were comparable at time 0 and then decreased over 6 and 12 hours of perfusion in the defatting group, reaching lower levels than the control group. The bar represents the median and error bars the IQR. (B) At the histological assessment of MaS on H & E staining, a similar trend was observed. (C) Defatted livers were more depleted of glycogen at time 0. However, their levels increased, already surpassing the control group at 6 hours of perfusion. The areas positive for PAS were constant for the control group throughout the perfusion. The dots represent individual organs at the time points listed and the line indicates the median of the values for each group. (D) An image from a histologically moderately steatotic liver that received the defatting cocktail and (E) presented with a significant reduction in macrovesicular steatosis already at 6 hours of perfusion. (G) The presence of MiS in the same liver at time 0 is seen, (H) as is the change at 6 hours. (F) An image from a mildly steatotic liver from the defatting group with an important depletion of glycogen stores, (I) replenishing its stores over 6 hours of perfusion. Comparisons between groups were made with the Mann‐Whitney U test. *Statistical significance at *P* < 0.05.

Each experimental group initially had 1 liver histologically classified as severely steatotic, 2 as moderately steatotic, 1 as mildly steatotic, and 1 as nonsteatotic. Changes between categories throughout the perfusion and the presence of MiS are presented in Tables 2 and 3. Positive PAS staining of parenchymal areas was constant along the perfusion for the control group, whereas the defatting group presented with a trend of increase mainly during the initial 6 hours of perfusion (Fig. 2).

**Table 2:** Steatosis Assessment Analysis

Case	Donor Type	CIT, minutes	MaS*			MiS		
			Time 0	Time 6	Time 12	Time 0	Time 6	Time 12
Defatting Group								
1	DBD	823	Moderate	Moderate	Mild	Present	Present	Present
2	DCD	729	Moderate	Moderate	Mild	Present	Present	Present
3	DBD	700	Mild	None	None	Present	Present	Present
4	DBD	754	Severe	Moderate	Moderate	Present	Present	Present
5	DCD	820	None	None	None	Present	Present	Present
Control Group								
1	DBD	520	Mild	Mild	Mild	Present	Present	Present
2	DCD	740	Moderate	Moderate	Moderate	Present	Present	Present
3	DCD	800	None	None	None	Present	Present	Present
4	DBD	723	Severe	Severe	Severe	Absent	Absent	Absent
5	DBD	735	Moderate	Moderate	Moderate	Present	Present	Present

* The severity of MaS was assessed in accordance with the parenchymal hepatocytes that had fatty changes: none, <5%; mild, 5%‐30%; moderate, >30%‐60%; and severe, >60%. These are the results of MaS assessment on H & E–stained definitive paraffin sections, performed by 2 independent pathologists at our center blinded to the study’s groups, using the parameters specified in the Patients and Methods section. Results from frozen sections evaluated externally for clinical purposes are available in Supporting Table 2 and were not included in the study’s analysis.

**Table 3:** Steatosis Rates Variation Analysis

Parameter	Defatting (n = 5)	Control (n = 5)	*P* Value
T‐TGs analysis			
T‐TG, mg/g of liver			
Time 0	2.7 (1.9‐3.5)	3.3 (1.9‐3.7)	0.84
Time 6	1.6 (0.9‐2.6)	3.2 (1.7‐3.6)	**0.03**
Time 12	2.0 (1.2‐2.6)	3.1 (1.6‐3.4)	**0.02**
ΔT‐TG, %			
Time 0 to 6	38 (23‐51)	7 (3‐10)	**0.003**
Time 0 to 12	30 (21‐35)	10 (6‐13)	**0.002**
Histological assessment analysis, %			
MaS			
Time 0	31 (10‐58)	40 (7‐65)	0.82
Time 6	15 (5‐35)	30 (7‐65)	0.31
Time 12	15 (5‐22)	40 (7‐65)	0.13
ΔMas			
Time 0 to 6	40 (17‐50)	0 (0‐12)	**0.02**
Time 0 to 12	50 (15‐60)	0 (0‐12)	**0.005**

NOTE:

Bolded *P* values are statistically significant. ΔT‐TG and ΔMaS are related to the variation in the figures between beginning and end of the period divided by the beginning.

### Lipid Metabolism Modulation and Defatting

The levels of T‐TG dropped by 38% (23%‐51%) in the defatting group and 7% (3%‐10%) in the control group over 6 hours (*P* = 0.003), and 30% (21%‐35%) in the defatting group versus 10% (6%‐13%) in the control group over 12 hours (*P* = 0.002), in comparison with time 0. MaS rate of defatted livers decreased by 40% (17%‐50%) over 6 hours and 50% (15%‐60%) over 12 hours; there were no changes in the control group (*P* = 0.02 and *P* = 0.005, respectively; Fig. 2; Table 3).

**Figure 3:**
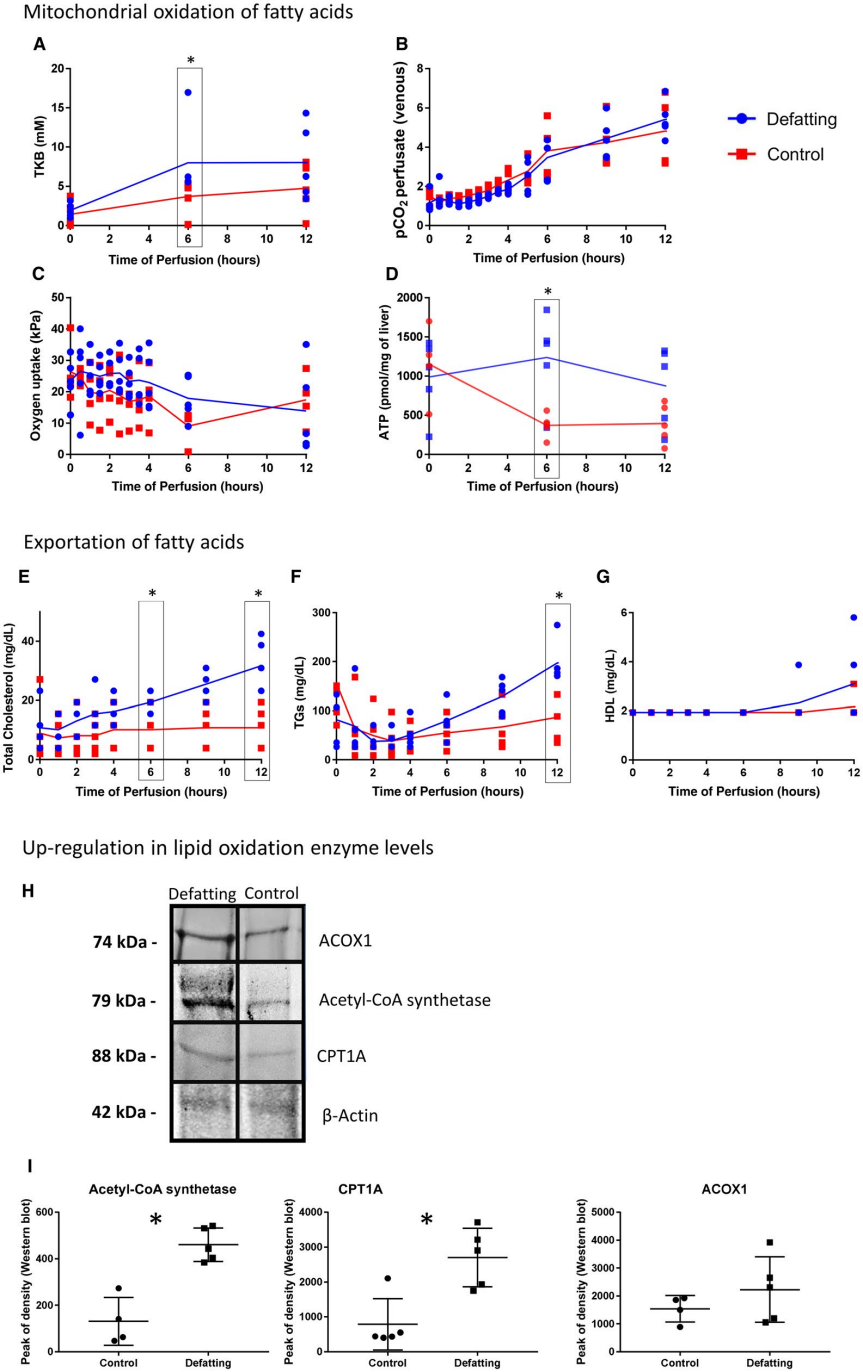
Metabolic pathways for elimination of intracellular TGs. (A‐D) Enhanced mitochondrial oxidation of FAs results in (A) increased ketogenesis throughout the perfusion. However, it was more pronounced in the treatment group. (B) Mitochondrial respiration assessed by PCO_2_ production increased steadily for both experimental groups, and (C) the oxygen uptake was higher for the initial 3 hours in the defatting group and then decreased slightly. (D) This change was associated with increased ATP replenishing within the initial 6 hours in the defatting group and then decreased steadily, maintaining higher concentrations than the control group. (E‐G) In addition, there was an increase in exportation of FAs. (E) The levels of total cholesterol in the perfusate increased faster in defatted livers than did the (F) levels of TGs. (G) HDL cholesterol levels were below the detection range and started to increase slowly after 6 hours of perfusion. (H,I) In‐gel fluorescent protein stain normalized to the total protein was performed for acetyl‐CoA synthetase, CPT1A, and peroxisomal ACOX1. Experiments were performed (n = 5), and (H) representative figures are presented. (I) The peak of intensity in densitometric analysis at the specific protein band was compared between the groups. In all panels, the dots represent individual organs at the time points listed and the line indicates the median of the values for each group. Comparisons between groups were made with the Mann‐Whitney U test. *Statistical significance at *P* < 0.05.

Ketogenesis was enhanced in the defatting group until 6 hours (*P* = 0.008). ATP levels were similar at time 0 and then increased significantly until 6 hours of perfusion for the defatting group and decreased for the control group (*P* = 0.01 and *P* = 0.008, respectively; Fig. 3).

Median P‐TG fell over the first 2 hours. Following this, it increased significantly until 6 hours in the defatting group (*P* = 0.04) but not in the control (*P* = 0.59). The increase in P‐TG levels showed a correlation with the drop in T‐TG between time points 0 to 6 hours (*r* = –0.58; *P* = 0.08) and 0 to 12 hours (*r* = –0.59; *P* = 0.07). There was no correlation between median P‐TG levels and ALT concentrations in the perfusate (*r* = 0.18; *P* = 0.65).

The levels of total cholesterol followed the same pattern and were higher in the defatting group at 6 (*P* = 0.02) and 12 hours of perfusion (*P* = 0.003). HDL cholesterol was below the limit of detection of the laboratory until 6 hours of perfusion (1.9 mg/dL) and started to increase thereafter; however, it did not achieve a statistically significant difference at 12 hours of perfusion (*P* = 0.28). Protein in‐gel staining revealed that defatted livers had at 6‐hour perfusion a 3.0‐fold increase in the protein levels of acetyl‐CoA synthetase, 1.4‐fold in ACOX1, and 6.0‐fold in CPT1A (Fig. 3).

### Liver Viability, Hepatocellular Metabolic Functional Parameters, and Biomarkers

Lactate levels were comparable at time 0, and then they decreased significantly until 12 hours of perfusion in the treatment group but not in the control group (*P* = 0.04 and *P* = 0.22, respectively). Dynamic changes in the lactate metabolism, as assessed by the AUC, showed that, considering both groups, smaller lactate AUC was associated with higher total perfusion flows (*r* = –0.84; *P* = 0.002), increased urea production (*r* = –0.73; *P* = 0.02), higher bile pH at 6 hours (*r* = –0.88; *P* = 0.02), increased release of P‐TG (*r* = –0.90; *P* < 0.001), lower expression of 4‐HNE (*r* = 0.62; *P* = 0.05), and a trend for higher bile production (*r* = –0.58; *P* = 0.08). The defatting group demonstrated a trend of shorter time to peak lactate and time to achieve lactate <2.5 mmol/L, which were correlated with positive metabolic factors. Further details are provided in Supporting Fig. 1.

All treated livers reached our viability criteria and therefore would be considered transplantable based solely upon these criteria. From the control group, only 2 organs recovered functional parameters that would be considered transplantable (*P* = 0.04). Achievements of the parameters of the criteria are presented in Supporting Table 3.

Defatted livers produced more urea over the initial 6 hours (*P* = 0.03). There was a strong negative correlation between lactate and urea levels at 6 hours (*r* = –0.82; *P* = 0.004) and 12 hours (*r* = –0.77; *P* = 0.009). Increased P‐TG correlated with an increased production of urea at 6 hours (*r* = 0.87; *P* = 0.001) and 12 hours (*r* = 0.63; *P* = 0.05). Median perfusate glucose increased from time 0 to 2 hours in the defatting group (*P* = 0.10) and then decreased toward 12 hours (*P* = 0.08). Those levels were constant for the control group. Metabolic parameters of the livers are presented in Fig. 1. Electrolytes and other biochemistry parameters are presented in Supporting Fig. 2.

The viable livers from the control group released less median (IQR) total cholesterol (17.4 [15.5‐19.3] versus 30.9 [23.2‐38.7] mg/dL; *P* = 0.02) and P‐TG (110.7 [88.6‐132.9] versus 177.1 [177.1‐186.0] mg/dL; *P* = 0.048) at 12 hours and produced less ATP (400 [390‐410] versus 1417 [1138‐1453] pmol/mg; *P* = 0.03) and developed a higher expression of 4‐HNE (IRS, 2 [2‐2] versus 1 [1‐1]; *P* = 0.02) at 6 hours in comparison with defatted livers.

### Biliary Injury and Function

Cumulative bile production was higher for the defatting group at the 6‐hour (*P* = 0.03) and 12‐hour perfusion time points (*P* = 0.008). There was no statistically significant correlation between total cholesterol in the perfusate and cumulative bile production at 6 hours (*r* = 0.59; *P* = 0.07) or 12 hours of NMP (*r* = 0.59; *P* = 0.07).

Bile quality, as assessed by the bile pH measured at 12 hours, was also higher in the defatting group (7.8 [7.7‐8.0] versus 7.3 [7.1‐7.6]; *P* = 0.03). GGT levels in the perfusate increased significantly from time 0 to 12 hours of perfusion in the control group (*P* = 0.04). Details are presented in Fig. 1.

### Hepatocellular Injury, Oxidative Damage, and Activation of Immune Cells

**Figure 4:**
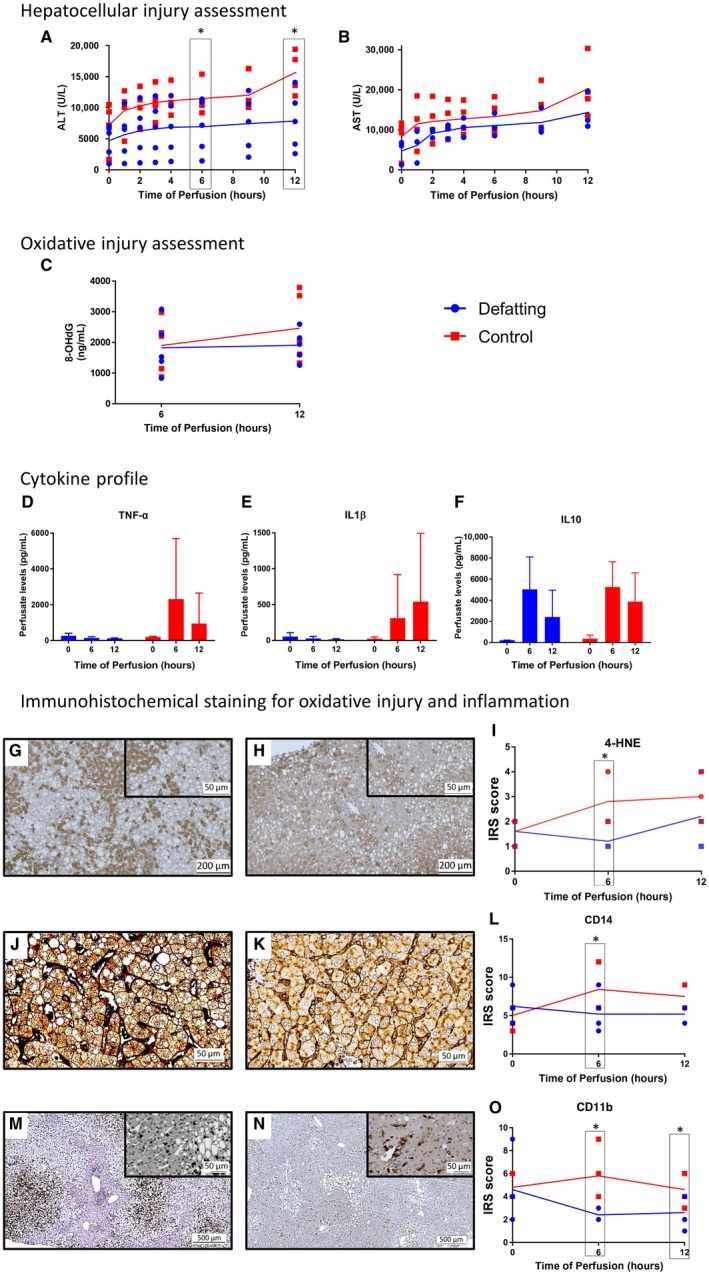
Assessment of hepatocellular and oxidative injury and activation of the immune cells. (A) ALT levels released in the perfusate were investigated as a marker of hepatocellular injury. Levels flattened along the perfusion in the defatting group and reached higher figures in the control group. (B) AST levels followed a similar trend. Markers of oxidative injury were also explored. (C) Nuclear cell damage assessed by 8‐HOdG released in the perfusate showed stable levels in the defatting group and a trend to increase in the control group. (D‐F) The cytokine profile was investigated, and perfusate concentration of the proinflammatory cytokines (D) TNF‐α and (E) IL1β decreased along the perfusion in the defatting group and increased in the control group. (F) The anti‐inflammatory IL10 increased in both groups at 6 hours and then it decreased faster in the defatted livers. Immunohistochemical analysis was performed for markers of oxidative injury and activation of an inflammatory response. (G) A moderate reaction of the staining committing 10%‐50% of the hepatocytes as assessed by the IRS. (H) After 6 hours of defatting, a predominantly mild staining reaction in 10%‐50% of the hepatocytes is seen. (I) The trends for each experimental group along the perfusion are presented. Similarly, tissue expression of (J,K) CD14 and (M,N) CD11b for the defatting group decreased from a time of 0 to a time of 6 hours of perfusion. (L) The changes over time for CD14 are indicated as are (O) the changes in tissue expression of CD11b. Scales are provided on the bottom of each panel. In all panels, the dots represent individual organs at the time points listed and the line indicates the median of the values for each group. Comparisons between groups were made with the Mann‐Whitney U test. *Statistical significance at *P* < 0.05.

Perfusate ALT levels were lower in the defatted livers at 12 hours of perfusion (*P* = 0.049; Fig. 4). Perfusate 8‐HOdG levels were similar at the 6‐hour time point followed by a general increase until 12 hours in the control group and a stabilization of levels in the defatting group (*P* = 0.10). Immunohistochemical analysis for 4‐HNE demonstrated similar initial levels at the beginning of the perfusion followed by an increase in the control group and a decrease in the defatting group at 6 hours (*P* = 0.02). There was a strong negative correlation between P‐TG and 4‐HNE expression (*r* = –0.87; *P* = 0.001) at 6 hours of perfusion.

The activation of the Kupffer cells and neutrophils in liver tissue, as assessed by immunohistochemical analysis (CD14 and CD11b, respectively), was similar between groups at time 0, and then, it increased for the control group, reaching higher scores at 12 hours (*P* = 0.046 and *P* = 0.045, respectively). The cytokine profile showed a decrease in the perfusate levels of the inflammatory TNF‐α from time 0 to 6, and from 0 to 12 hours in the defatting group. IL1β also decreased over the course of the perfusions in the treatment group. For both markers, the levels in the control were either flat or increased. The anti‐inflammatory IL10 increased in both groups from the beginning until a time of 6 hours, and then, it decreased faster in the defatting group (Fig. 4).

Of note, similar enhancement in the lipid metabolism leading to a decrease in T‐TG, via increased oxidation of FAs and solubilization in the perfusate, along with improved biliary function and lower hepatocellular injury, was seen in a subanalysis of organs deemed mildly steatotic and nonsteatotic on histology. Results are presented in Supporting Table 4.

## Discussion

Steatosis has become the leading reason for surgeons to decline donor livers for transplantation, which, in turn, is worsening the growing discrepancy between organ availability and the increasing number of patients on transplant waiting lists.^14^, ^15^ We have shown that the delivery of a pharmacological intervention during NMP was able to decrease the fat content of whole human livers within 6 hours. This effect was driven by enhanced lipid metabolism, as increased oxidation of lipids and exportation to the perfusate. Importantly, this enhanced lipid metabolism increased the metabolic support to the organ, improving its functional recovery. It was associated with enhanced mitochondrial functioning, decreased vascular resistances, and reduced markers of hepatocellular injury and inflammation with improved biliary function. These parameters are suggested as being indicative of transplantability for extended criteria donor (ECD) organs undergoing NMP, according to the evidence available to date.^16^, ^17^ Therefore, manipulation of the hepatic lipid metabolism during NMP may defat steatotic organs and increase their functional recovery, potentially halting the harmful effects of reperfusion injury.

Previous in vitro studies with fat‐laden rat hepatocytes and hepatoblastoma cells demonstrated the feasibility of defatting cells with a combination of drugs over 48 hours.^6^, ^18^, ^19^ Thereafter, this defatting cocktail was delivered to whole steatotic rat livers during NMP, and the authors demonstrated a decrease in T‐TG of 50% within 3 hours.^6^ Interestingly, a decrease of 30% could be obtained with NMP alone over the same period.^6^ However, a recent study reporting results of 24 hours of perfusion of steatotic human livers could not replicate the same results with NMP alone.^5^ This raised concerns about variability in response between species and about how the effectiveness of the drugs could be compromised after the inherent ischemic injury that follows organ retrieval.

A description of the detailed pharmacological effects of the drugs used is out of the scope of this manuscript, but the effects of the drugs have recently been reviewed.^11^ The evidence for the safety of the individual components of the defatting agents is presented in Supporting Table 5. Although the authors believe that there is solid evidence supporting the safety of using all of the suggested drugs during NMP, there is a lack of clinical markers for some of the compounds; thus, a selection of those drugs undergoing clinical trials may be an option. Our group recently reported, for the first time, the in vitro effectiveness of these pharmacological agents in defatting primary human hepatocytes that were made fatty by supplementation with FAs.^10^ Apart from being nontoxic to these cells, the defatting process decreased intracellular hepatocyte triglycerides (TG) by 35% over 48 hours of incubation. These agents were also shown for the first time to not be toxic to nonparenchymal liver cells (intrahepatic endothelial cells and cholangiocytes).^10^

**Figure 5:**
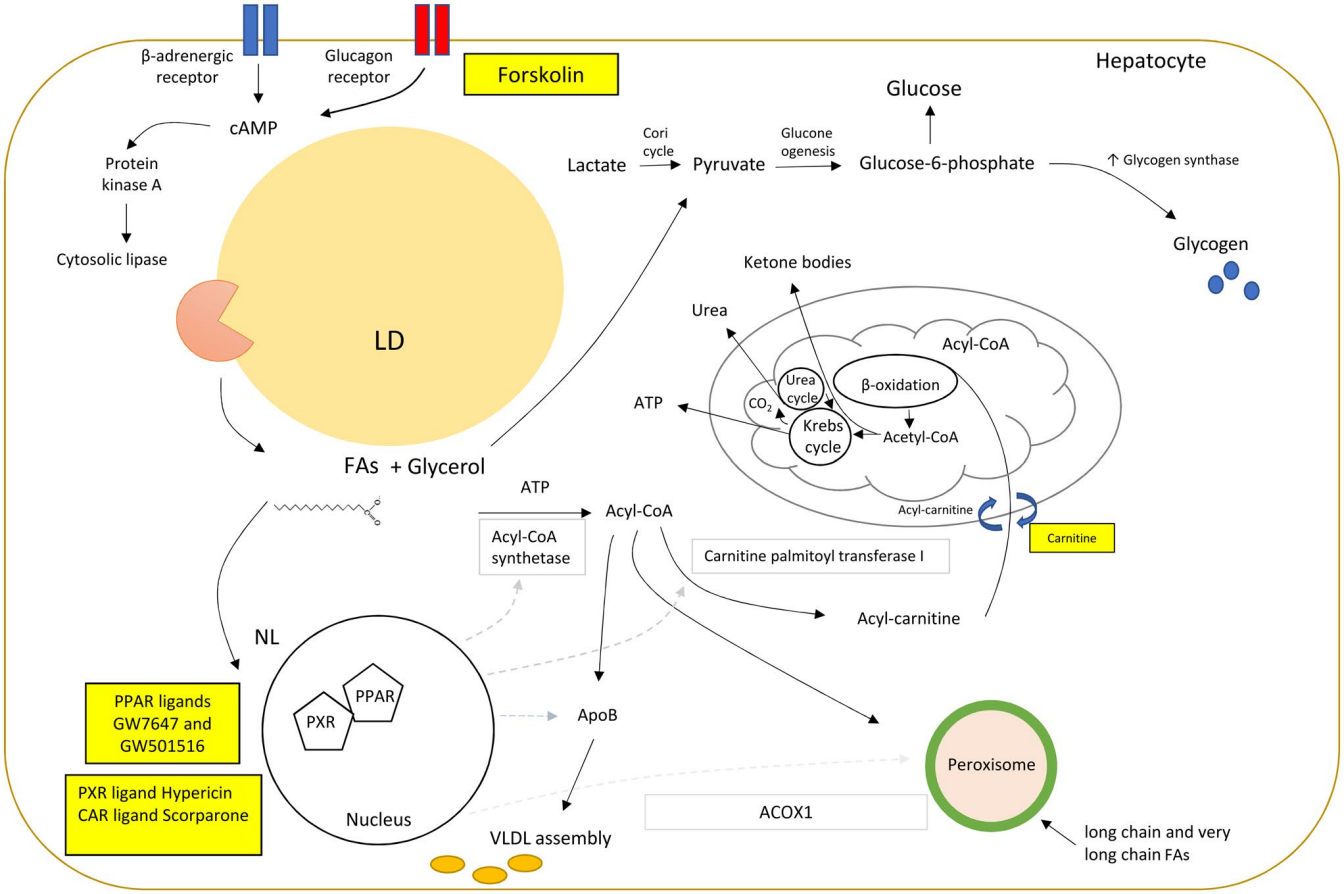
A suggested relationship between the defatting cocktail and intracellular lipolytic metabolic pathways. Forskolin activates the glucagon membrane receptors, which in turn stimulate the cAMP–protein kinase A pathway; thus, the cytoplasmic lipases are attracted to the surface of LDs. Glycerol and FFAs will then be released, serving not only as substrates for the cell metabolism but also as ligands to nuclear receptors (PPAR and liver X receptor), increasing the transcription of enzymes involved in the catabolism of FFAs in the mitochondria and peroxisome. Diverse cocktail drugs (GW7647, GW501516, hypericin, scorparone) also act as ligands to other nuclear receptors (PXRs and CARs), boosting the transcription of key enzymes. Cytosolic FA reacts with ATP, producing fatty acyl‐CoA. Acyl‐CoA in turn reacts with apolipoprotein B to generate lipoproteins to be exported from the cell and/or reacts with the hydroxyl group of carnitine via carnitine palmitoyltransferase I. Acyl‐carnitine is transported inside the mitochondria by a carnitine‐acyl‐CoA transferase, and a carnitine is transferred outside. Acyl‐CoA is processed by β‐oxidation. The acetyl‐CoA produced then will follow to ketogenesis or for complete oxidation via the Krebs cycle and the electron transport chain with the production of ATP. Increased production of aspartate and CO_2_ stimulate the urea cycle, increasing the production of urea. Alternatively, long chain and very long chain FFAs are also oxidized in the peroxisomes. Glycerol is a gluconeogenic precursor; it is converted to pyruvate, producing glucose‐6‐phosphate through the gluconeogenesis pathway. Glucose‐6‐phosphate will be released as glucose in the perfusate and stimulate the enzyme glycogen synthase, increasing the production of glycogen. The squares contain the specific enzymes that have an up‐regulation in the transcription as a consequence of this stimulus. Drugs and supplement used in the cocktail are presented in yellow squares.

In this study, we delivered the defatting cocktail to human donor livers exposed to extended periods of CIT. We have found that NMP supplemented with the defatting cocktail improves lipid metabolism, significantly decreasing T‐TG by 38% over a period of 6 hours, whereas NMP alone reduced it by 7% over the same time. Histologically, it corresponded to a decrease of 40% in MaS rate in the defatting group and there was no change in the control group. This defatting effect was associated with an enhanced mitochondrial function (β‐oxidation of FAs, mitochondrial respiration [higher oxygen uptake], ketogenesis, augmented urea cycle, and Krebs cycle activity with higher ATP synthesis) and increased exportation of intracellular TGs as lipoproteins in the perfusate. Mechanistically, it was represented by up‐regulation in the levels of key enzymes involved in intracellular lipid metabolism in comparison with control livers. Beneficial effects were also seen in glucose metabolism, where defatted livers released more glucose in the perfusate during the initial hours of perfusion. This enhanced gluconeogenesis is likely to be related to an increased availability of glycerol because of the augmented breakdown of lipids with higher production of glucose‐6‐phosphate. Glucose‐6‐phosphate, in turn, will generate the glucose released in the perfusate and activate the enzyme glycogen synthase promoting glycogenesis, in accordance with our findings.^20^ A diagrammatic summary is provided in Fig. 5.

In addition, modulation of the lipid metabolism halted hepatocellular injury as assessed by the diminished release of ALT in the perfusate. Tissue damage during reperfusion is primarily related to mitochondrial dysfunction with reactive oxygen species (ROS) production, oxidative stress, and a concomitant proinflammatory response. Accordingly, treated livers developed lower cellular DNA damage (8‐HOdG) by the end of the perfusion as well as lower rates of lipid peroxidation (4‐HNE), production of inflammatory cytokines, and activation of immune cells (CD14, CD11b). The former could also have resulted from the direct effects of the defatting constituents, such as PPARs.^21^, ^22^ Our findings corroborate those of previous in vitro studies, in which delivering the cocktail to fat‐laden rat hepatocytes diminished ROS concentration and cellular injury, as assessed by the cytosolic lactate dehydrogenase release into media.^18^

Ischemic cholangiopathy following DCD liver transplantation is of great clinical concern, and identification of high‐risk donor organs during NMP, prior to transplantation, would be a useful clinical tool.^23^ Watson et al. described how bile pH <7.5 during NMP is suggestive of biliary tree necrosis and can increase the risk of ischemic cholangiopathy after transplantation.^17^ Contrary to this finding, treated livers demonstrated bile pH levels higher than 7.5, whereas the converse was seen in control livers. Importantly, no correlation was found between greater bile production and the increase in total cholesterol in the perfusate, suggesting a beneficial effect of the cocktail itself on the biliary system. The mechanism involved needs further investigation. However, the protective role of defatting in lowering oxidative injury and tissue damage can be potentially correlated.

In accordance with in vitro studies in which hepatocyte viability improved, the defatting cocktail used in this study enhanced the functional recovery of donor livers during NMP.^18^, ^24^, ^25^ Urea is produced by the liver and results from an enzymatic reaction involving carbon dioxide and the ammonia derived from the deamination of proteins in the liver. The increased level of urea suggests increased urea synthesis in defatted livers.^19^, ^26^ Our previously published viability criteria assert that lactate levels <2.5 mmol/L within 4 hours is a major criterion for safe transplantation of ECD donor livers. However, it also considers other parameters of liver metabolism.^12^, ^16^ We have performed a separate analysis of the dynamic changes in lactate concentration over time. It showed a strong correlation between lactate metabolism kinetics (lower AUC, shorter time to peak lactate and the clearance from peak to 2.5 mmol/L) and other parameters of appropriate liver metabolism. These findings reinforce the significance of this marker in the context of defatting. The Cambridge group has advocated the use of transaminases, bile production, and bile quality as appropriate markers of organ viability.^17^ The discussion about optimal viability criteria during NMP is out of the scope of this paper, and another topic for discussion is whether the same criteria or machine perfusion technique is applicable for all organs (eg, DBD, DCD, or defatted livers). However, defatted livers fulfilled all those criteria that are currently considered indicative of adequate liver metabolism and, hence, would potentially be deemed transplantable.

The results obtained from employing our study protocol have shown that pharmacological targeting of lipid metabolism can defat steatotic human livers and improve their metabolic function within the relatively short time frame of 6 hours. Beyond this time point, all of the benefits observed with the defatting protocol appeared to be sustained. This observation suggests that the drugs had already been metabolized at the 6‐hour perfusion mark. At this point, either a second bolus of the cocktail could be considered to amplify its effects or the aim would be considered achieved and the organ could potentially be used for transplantation. Another point of discussion is the use of filters to remove solubilized lipids from the perfusate or even changing the perfusate, the intent of which is to potentially prevent lipotoxicity. However, in the context of NMP and defatting, we have not seen toxic effects of the FAs in organ functioning, tissue damage, or inflammatory response. Further studies will be needed to investigate its potential advantages.

Banan et al.^27^ showed a reduction of 10% in MaS over 8 hours of NMP for a discarded steatotic donor human liver with 80% MaS and a negligible reduction for a liver with 30% MaS, supplementing the perfusate with 10 mM of L‐carnitine. However, the variability between the 2 reported organs and the lack of a control group limits the interpretation of the results of this study. This is the first study designed specifically to deliver a defatting cocktail of drugs to steatotic human livers during NMP and to assess its impact on lipid metabolism. Importantly, all of these observations were made within a scenario of clinical organ donation encompassing livers originating from different donors (DCD and DBD) exposed to extended periods of CIT before receiving treatment. Moreover, it is important to highlight that all of these benefits were also seen for organs deemed mildly steatotic and nonsteatotic on histology that had MiS. This was demonstrated through a decrease in their levels of T‐TG along with the improved metabolic function and lower release of markers of hepatocellular injury.

Although our study has shown that ex situ pharmacological modulation of lipid metabolism of donor human livers enhances its function, we have not proven that the livers are transplantable. This concept clearly demands a pilot study to assess the defatting protocol in clinical transplantation. In the United Kingdom, histological assessment of donor liver steatosis is not performed routinely, and livers are discarded solely based on a surgical macroscopic assessment of steatosis. As a consequence, our groups included organs with various grades of steatosis, which was assessed at the histological level, with 2 livers having <5% MaS. However, they were equally distributed between groups. Of note, from the 3 livers deemed severely steatotic on a frozen section at an external center, 2 of them had no MaS on definitive paraffin sectioning. This discrepancy may be due to sampling issues, tissue preparation, or inconsistency in the assessment between pathologists. To prevent bias, all of the study’s analyses were performed with results from definitive paraffin sections that were assessed blindly by 2 pathologists at our center, applying the standardized criteria presented in the Patients and Methods section. Moreover, despite controlling for the donor type, CIT, and livers with similar degrees of steatosis, some other unfavorable factors were more predominant in the control group (higher GGT peak, higher liver weight, the oldest donor in the cohort), and the causes of death were not perfectly matched within donor type categories. Small differences between groups are an intrinsic limitation of research with discarded human livers; however, the use of human livers in research has the advantage of eliminating variability between species, the latter being a limitation of animal model studies. All livers had prolonged CIT, which, together with steatosis and other donor factors, explains the poor rescue of control livers based on metabolic parameters.

In addition, although we were unable to control the groups for the causes of hepatic steatosis—that would be based on the history of alcohol intake and the presence of risk factors for metabolic syndrome, such as diabetes mellitus and obesity—we believe this mimics real life. This is because the coexistence of these factors in the same donor makes it difficult to define a precise etiology at the time of the offer of a steatotic donor liver for transplantation. Mechanistically, the drugs increase the intracellular lipolysis via the cAMP–protein kinase A pathway and induce the transcription of enzymes involved in FAs catabolism in the mitochondria and peroxisomes, independently of insulin receptors. Thus, either the occurrence of insulin resistance, the hallmark of nonalcoholic fatty liver disease, or its absence should not affect the process.

In conclusion, we have shown that pharmacological modulation of lipid metabolism during NMP can promote defatting of whole human steatotic livers within 6 hours. More importantly, the enhanced lipid metabolism improved the metabolic status of the organs and their functional recovery, decreased vascular resistance, and reduced the expression of markers of reperfusion injury. Those findings support further clinical investigations and open a window of opportunity for better use of steatotic donor livers.
