# Supplementary material for: Manipulation of Lipid Metabolism During Normothermic Machine Perfusion: Effect of Defatting Therapies on Donor Liver Functional Recovery
Source: Liver Transpl. 2019 Jun 20;25(7):1007–22. doi: 10.1002/lt.25439 (PMC6618030; doi:10.1002/lt.25439)

**SUPPLEMENTARY MATERIAL**

**Manipulation of lipid metabolism during normothermic machine perfusion: Effect of defatting therapies on donor liver functional recovery**

Yuri L Boteon^1,2,3^, Joseph Attard^1,2,3^, Amanda PCS Boteon^1^, Lorraine Wallace^2,3^, Gary Reynolds^2,3^, Stefan Hubscher^1,4^, Darius F Mirza^1,2,3^, Hynek Mergental^1,2,3^, Ricky H Bhogal^5^, Simon C Afford^2,3^

**Affiliations:**

1. Liver Unit, Queen Elizabeth Hospital, University Hospitals Birmingham NHS Foundation Trust, Birmingham, United Kingdom

2. Centre for Liver and Gastrointestinal Research, Institute of Immunology and Immunotherapy, College of Medical and Dental Sciences, University of Birmingham, Birmingham, United Kingdom

3. National Institute for Health Research (NIHR) Birmingham Biomedical Research Centre, University of Birmingham and University Hospitals Birmingham NHS Foundation Trust, United Kingdom

4. Department of Cellular Pathology, Queen Elizabeth Hospital, University Hospitals Birmingham NHS Foundation Trust, Birmingham, United Kingdom

5. The Royal Marsden, Department of Academic Surgery, Fulham Road, Chelsea, London, SW3 6JJ

**Table of content**

**1. Supplementary methods**

**1.1. Liver perfusion procedure**

**1.2. Sampling protocol**

**1.2.1. Liver biopsies**

**1.2.2. Perfusate samples**

**1.2.3. Bile samples**

**1.3. Data collection protocol**

**1.4. Fluorescent protein gel stain**

**1.5. Immunohistochemical staining**

**1.6. Antibodies list**

**1.7. Reagents list**

**2. Supplementary results**

**2.1. Table S1**

**2.2. Table S2**

**2.3. Tale S3**

**2.4. Table S4**

**2.5 Table S5**

**2.6. Figure S1**

**2.7. Figure S2**

**1.0. Supplementary methods**

**1.1. Liver perfusion procedure**

The liver was flushed via portal vein (PV) and hepatic artery (HA) with 2 litres of 5% glucose, transferred to the reservoir of the disposable and then perfusion was started. Liver Assist (Organ Assist, Groningen, Netherlands) was the device used for the perfusions. Temperature was set initially at 20oC increasing gradually to 37oC within the first 30 minutes. Initial pressure on the HA was 30 mmHg and 5 mmHg on the PV. Both were raised steadily with incremental increases in temperature reaching 60 mmHg and 10 mmHg respectively at 30 minutes. Oxygen supply was adjusted to achieve partial pressure of 20-30 kPa in the perfusate on the arterial circuit.

**1.2. Sampling protocol**

***1.2.1. Liver biopsies***

Core needle liver biopsies using disposable modified Menghini needle (15G) were taken before commencing the perfusion, at time points 6-hour and 12-hour of perfusion for all livers. Two biopsies not far 1 cm from each other were taken at each time point. One was stored in formalin and the other immediately snap-frozen in liquid nitrogen for further analysis.

***1.2.2. Perfusate samples***

The perfusate was sampled every 30 minutes throughout the first 4 hours of perfusion and then at 6-hour, 9-hour and 12-hour of perfusion for all livers.

At each time point approximately 1 millilitre (mL) of perfusate was analysed immediately using the Cobas b 221 point of care system (Roche Diagnostics, USA) blood gas analyser. The partial pressures of oxygen (O_2_), and carbon dioxide (CO_2_), pH, concentrations of base excess, bicarbonate, O_2_ saturation, haemoglobin, haematocrit, sodium, potassium, chloride, calcium, glucose and lactate were measured. Four mL of perfusate were then freshly collected in serum separators tubes for biochemistry analysis at the hospital clinical laboratories. Six mL were centrifuged at 10,000 rotations per minute for 2 minutes and the supernatant collected and snapped-frozen for further analysis.

***1.2.3. Bile samples***

Bile production was collected into a Jackson-Pratt drain accoupled to the pipe connected with the bile duct. The drain was empty at 2-hour, 4-hour, 6-hour, 8-hour and 12 hours of perfusion for all livers.

At each time point the total volume of bile produced was recorded and the bile analysed using the Cobas b 221 point of care system (Roche Diagnostics, USA) blood gas analyser. Four 4 mL were collected into cryovials and snapped-frozen for further analysis.

**1.3. Data collection protocol**

Parameters for the arterial and venous perfusion units (flows, pressure, resistance and temperature) showed on the device’s display were registered every 30 minutes throughout the first 4 hours of perfusion and then at 6-hour, 9-hour and 12-hour of perfusion for all livers.

**1.4. Fluorescent protein gel stain**

Equal amounts of frozen liver tissue were homogenised in lysing buffer solution (Tris, 2% SDS [sodium dodecyl sulfate], 2 mM EDTA [Ethylenediaminetetraacetic acid], ph=9.0) at 100 mg/mL using the Precellys 24 tissue homogeneiser (Bertin instruments, Montigny-le-Bretonneux France). Protein concentration was determined using the bicinchoninic acid (BCA) protein assay (23225; Thermo Scientific Pierce, Waltham, MA, USA) and final concentration equalised to 10 mg/mL. Samples were diluted in a 1:1 rate with 2x Laemmli sample loading buffer (1610737; Bio-Rad, Hercules, CA, USA) 5% 2-mercaptoethanol. Thereafter 20 µL of sample total protein were loaded in each well of a polyacrylamide gel (Sodium dodecyl sulfate-polyacrylamide gel - SDS-PAGE) (4561045; Bio-Rad, Hercules, CA, USA) on the Criterion Dodeca gel apparatus (Bio-Rad) and protein bands were separated by electrophoresis at 200V for 30 minutes in running buffer (1610732; Bio-Rad). Precision Plus Protein™ WesternC™ Blotting Standards (Bio-Rad) was used as a protein ladder. An alcohol solution (50% isopropanol, 5% acetic acid prepared in ultrapure water), was used to fix protein bands on the matrix and the gel was incubated for 1 hour with the primary antibody (see antibody list). Following the gel was washed in PBS (Phosphate-buffered saline) plus 0.1% Tween 20 and then incubated with the secondary fluorescent antibody for an additional 1 hour (see antibody list). β-Actin antibody was used as a loading control to confirm that samples were equally loaded and the effectiveness of the electrophoresis and imaged using a secondary fluorescent antibody. Gels were imaged with the ChemiDoc MP imager (Bio-Rad, Hercules, CA, USA) using specific protocols for the antibodies used based upon their excitation and emission specifications. Images were analysed using the ImageLab software version 6.0 (Bio-Rad). Background was subtracted from all lanes and the peak of intensity in densitometric analysis at the specific protein band compared between the groups.

**1.5. Immunohistochemical staining**

Immunohistochemistry was performed on paraffin embedded tissue sections of 4 μm thickness using the ImmPRESS^TM^ Excel Staining kit (MP-7601 and MP-7602; Vector Laboratories, Burlingame, CA, USA). The sections were deparaffinized in 3 baths of xylene for 3 minutes and rehydrated in a similar series of ethanol. Microwaving antigen retrieval was performed in Tris-based solution (H-3301; Vector Laboratories), pH 9, for 25 minutes. The sections were washed using Dako wash buffer (S3006; Dako Agilent, Santa Clara, CA, USA). Quenching of endogenous peroxidase activity was done via incubation in BLOXALL™ Blocking Solution (SP-6000; Vector laboratories) for 10 minutes and blocked with 1x casein for 10 minutes (SP-5020; Vector Laboratories). Sections were then incubated with the primary antibodies diluted in 1x casein (see antibody list for specific dilutions used) for 1 hour. After a second wash tissue sections were incubated with an amplifier antibody (goat anti-mouse IgG or goat anti-rabbit IgG) ready-to-use (Vector laboratories) for 15 minutes. A new wash was performed, and the tissue sections incubated with ImmPRESS^TM^ Excel reagent for 30 minutes. The positive immuno-reactivity was revealed by incubation with a mixture of ImmPACT™ DAB EqV Reagent 1 with Reagent 2 (Vector laboratories). Tissue sections were counterstained with Mayer’s haematoxylin. Finally, sections were dehydrated in a series of ethanol and xylene. No primary negative controls were obtained by replacing primary antibody for equal volume of goat serum and isotype negative controls by replacing the primary antibody for an isotype control antibody.

All sections were wholly digitalized using the slide scanner Axio Scan.Z1 (Carl Zeiss, Oberkochen, Germany) and the images for the study selected using the ZEN image analysis software.

**1.6. Antibodies list**

**Immunohistochemical staining**

- Anti-4-hydroxynonenal (4-HNE) antibody (ab46545; Abcam, Cambridge, MA, USA) rabbit polyclonal IgG at 1:200 dilution.
- Anti-Cluster of differentiation 14 (CD14) antibody (ab36595; Abcam, Cambridge, MA, USA) mouse monoclonal at 1:500 dilution.
- Anti-Cluster of differentiation 11b (CD11b) antibody (ab52478; Abcam, Cambridge, MA, USA) rabbit monoclonal at 1:200 dilution.
- Rabbit IgG isotype control antibody (ab172730; Abcam, Cambridge, MA, USA) monoclonal at 1:200 dilution.
- Mouse IgG isotype control antibody, clone MG3-35 (catalogue number 401302; BioLegend, San Diego, CA, USA) unspecific at 1:500 dilution.

**Protein gel staining**

**Primary antibodies**

- Anti-Acetyl CoA synthetase antibody (ab133664; Abcam, Cambridge, MA, USA) rabbit monoclonal IgG at 1:1,000 dilution.
- Anti-ACOX1 antibody (ab184032; Abcam, Cambridge, MA, USA) rabbit monoclonal IgG at 1:1,000 dilution.
- Anti-CPT1A antibody (ab83862; Abcam, Cambridge, MA, USA) rabbit polyclonal IgG at 1:1,000 dilution.
- Anti-β-Actin antibody (A5441; Sigma-Aldrich, St. Louis, MO., USA) mouse monoclonal IgG at 1:1,000 dilution.

**Secondary antibodies**

- Sheep anti Rabbit IgG:DyLight®488 (STAR36D488GA; Bio-Rad, Hercules, CA, USA) polyclonal IgG at 1:1,000 dilution.
- Goat anti Mouse IgG:DyLight®800 (STAR117D800GA; Bio-Rad, Hercules, CA, USA) polyclonal IgG at 1:1,000 dilution.

**1.7. Reagents list**

All drugs employed in the experiments were bought from Sigma-Aldrich, St. Louis, MO., USA. The corresponding catalogue numbers are: Forskolin-F6886; GW7647-G6793; Hypericin-56690; Scoparone-254886; GW501516-SML1491; Visfatin-SRP4908; and, L-carnitine-C0283. General laboratory consumables used were mostly bought from Sigma-Aldrich.

**2.0. Supplementary results**

**2.1. Table S1: Normothermic machine perfusion fluid constitution.**

| 3 units of group O Rhesus-negative donor packed red blood cells |
| --- |
| 1000 mL of 5% w/v human albumin solution (Alburex 5, CSL Behring GmbH, Germany) |
| 10,000IU heparin (Wockhardt, UK) |
| 30 mL sodium bicarbonate 8.4% (B. Braun Medical Limited, UK) |
| 10 mL calcium gluconate 10% |
| 500 mg vancomycin (Wockhardt, UK) |
| 60 mg gentamicin (Cidomycin, Sanofi, UK) |
| 50 mL of 10% v/v Aminoplasmal (B.Braun Medical Limited, UK) |
| 0.2 mL Cernevit (Baxter Healthcare Ltd., UK) |
| 0.1 mg phytomenadione (Konakion, Roche Products Ltd, UK) |
| Epoprostenol (Flolan, GlaxoSmithKline, UK, 2 µg/ml) continuous infusion at 4 mL/hour |

**Abbreviations:** UK- United Kingdom.

| **Liver number** | **CONTROL 1** | **CONTROL 2** | **CONTROL 3** | **CONTROL 4** | **CONTROL 5** | **DEFAT 1** | **DEFAT 2** | **DEFAT 3** | **DEFAT 4** | **DEFAT 5** |
| --- | --- | --- | --- | --- | --- | --- | --- | --- | --- | --- |
| **Donor information** | | | | | | | | | | |
| Age (years) | 81 | 48 | 49 | 52 | 41 | 51 | 69 | 54 | 52 | 44 |
| Donor type | DBD | DCD | DCD | DBD | DBD | DBD | DCD | DBD | DBD | DCD |
| Gender | Male | Male | Male | Female | Female | Male | Male | Male | Female | Female |
| Height (cm) | 173 | 170 | 203 | 172 | 165 | 174 | 193 | 175 | 169 | 155 |
| Body weight (kg) | 76 | 95 | 115 | 75 | 95 | 81 | 101 | 98 | 86 | 70 |
| Body mass index (kg/m^2^) | 25 | 33 | 28 | 25 | 35 | 37 | 27 | 32 | 30 | 29 |
| Donor risk index | 2.1 | 2.0 | 2.1 | 2.0 | 1.7 | 2.0 | 3.0 | 1.8 | 2.0 | 2.6 |
| UK donor liver index | 1.5 | 2.0 | 1.3 | 1.1 | 0.8 | 1.0 | 1.8 | 1.2 | 0.9 | 1.8 |
| ET donor risk index | 2.0 | 2.0 | 2.6 | 2.4 | 2.0 | 1.8 | 3.4 | 1.9 | 1.8 | 2.8 |
| Peak ALT (IU/L) | 39 | 74 | 177 | 104 | 335 | 76 | 67 | 110 | 30 | 65 |
| Peak GGT (IU/L) | 31 | n/a | 137 | 402 | 355 | 31 | 71 | 150 | 38 | 269 |
| Days on ventilator | 1 | 5 | 4 | 3 | 2 | 2 | 2 | 3 | 2 | 4 |
| Co-morbidities and risk history | Hypertension | Heavy drinker | Light drinker | Heavy drinker, depression | Hypertension | Diabetes (type 2) | Diabetes (type 2), moderate drinker | Diabetes (type 2), Smoker | Hypothyroidism, light drinker | Depression, ulcerative colitis |
| Cause of death | Trauma | Hypoxic brain damage | Hypoxic brain damage | Intracranial haemorrhage | Hypoxic brain damage | Intracranial haemorrhage | Intracranial haemorrhage | Intracranial haemorrhage | Intracranial haemorrhage | Hypoxic brain damage |
| **Organ information** | | | | | | | | | | |
| Liver weight (grams) | 1699 | 1852 | 2139 | 2130 | 2317 | 1768 | 2330 | 2394 | 1874 | 1694 |
| Donor warm ischaemia time (min) | n/a | 17 | 15 | n/a | n/a | n/a | 13 | n/a | n/a | 14 |
| Cold ischaemic time (minutes) | 520 | 740 | 800 | 723 | 735 | 823 | 729 | 700 | 754 | 820 |
| Reason for clinical rejection* | Steatosis | Steatosis | Steatosis (severe on biopsy) | Steatosis | Steatosis | Steatosis | Steatosis | Steatosis | Steatosis (severe on biopsy¥) | Steatosis (severe on biopsy) |
| **Perfusion characteristics** | | | | | | | | | | |
| Lactate (mmol/L) |  |  |  |  |  |  |  |  |  |  |
| Highest | 14.7 | 10.3 | 13.9 | >20.0 | 17.7 | 11.6 | 11.0 | 9.9 | 12.7 | 20.0 |
| Lowest | 1.6 | 0.8 | 0.7 | 11.8 | 11.6 | 1.9 | 0.9 | 0.2 | 1.8 | 0.4 |
| Last | 1.9 | 2.7 | 1.0 | >20.0 | 13.4 | 1.9 | 0.9 | 0.3 | 1.8 | 0.4 |
| Total Bile production (mL) | 6.0 | 40.0 | 8.0 | 0 | 38.0 | 70 | 40.0 | 31.0 | 40.0 | 30.0 |
| Median Arterial flow (mL/min) | 144 | 286 | 343 | 242 | 616 | 256 | 260 | 364 | 359 | 377 |
| Median Portal vein flow (mL/min) | 871 | 1103 | 1283 | 902 | 1020 | 1185 | 1316 | 1454 | 1079 | 1454 |
| Median liver mass perfusion (mL/g/min) | 0.6 | 0.7 | 0.8 | 0.5 | 1.0 | 0.8 | 0.7 | 0.8 | 0.8 | 1.1 |
| Viability achievement | No | Yes | Yes | No | No | Yes | Yes | Yes | Yes | Yes |

**2.2. Table S2:** **Donor demographics, liver features and perfusion parameters.**

**Abbreviations:** Defat- Defatting group; DBD- Donation after brain death; DCD- Donation after circulatory death; BMI- Body mass index; ET- Eurotransplant; WIT- Warm ischaemic time; CIT- Cold ischemic time; ALT- Alanine aminotransferase; GGT- Gamma-glutamyltransferase; n/a- not available or not applicable. *= whenever available, the histological grade of steatosis on frozen section biopsy done prior to the organ being discarded for transplantation was recorded; ¥= this result was not known before start perfusion.

**2.3. Table S3:** **Viability criteria achievement by the livers in each group.**

| **Criteria** | **Defatting (*n*=5)** | **Control (*n*=5)** |
| --- | --- | --- |
| Lactate clearance (≤2.5 mmol/L) | 5 (100%) | 2 (40%) |
| Ph >7.3 perfusate | 4 (80%) | 2 (40%) |
| Glucose metabolism | 5 (100%) | 4 (80%) |
| HA flow (>150 mL/ min) | 5 (100%) | 4 (80%) |
| PV flow (>500 mL/ min) | 5 (100%) | 5 (100%) |
| Homogeneous perfusion/ soft parenchyma | 5 (100%) | 5 (100%) |
| Bile production | 5 (100%) | 4 (80%) |
| Viable liver | 5 (100%) | 2 (40%) |

**Abbreviations:** HA- hepatic artery; PV- portal vein.

| **2.4. Table S4: Subanalysis of donor livers with None or Mild macrovesicular steatosis on histology. Donor demographics, liver, perfusion parameters and metabolic parameters of the organs.** | | |
| --- | --- | --- |
| **Characteristic** | **Defatting (*n*=2)** | **Control (*n*=2)** |
| **Donor information** | | |
| Age, years | 49 (44-54) | 65 (49-81) |
| DCD livers | 1 (50%) | 1 (50%) |
| Sex, male | 1 (50%) | 2 (100%) |
| Height (cm) | 165 (155-175) | 188 (173-203) |
| Bodyweight (kg) | 84 (70-98) | 95 (76-115) |
| Body mass index (kg/m^2^) | 31 (29-32) | 27 (25-28) |
| Donor risk index | 2.2 (1.8-2.6) | 2.1 (2.0-2.1) |
| UK donor liver index | 1.5 (1.1-1.8) | 1.4 (1.3-1.5) |
| ET donor risk index | 2.3 (1.8-2.8) | 2.3 (2.0-2.6) |
| Peak ALT (IU/L) | 87 (65-110) | 39 |
| Peak GGT (IU/L) | 209 (150-269) | 84 (31-137) |
| Days on ventilator | 3 (3-4) | 2 (1-4) |
| **Liver characteristics** | | |
| Liver weight (grams) | 2044 (1694-2394) | 1919 (1699-2139) |
| Donor warm ischaemia time (minutes) | 14 | 13 |
| Cold ischaemia time (minutes), DCD | 820 | 920 |
| Cold ischaemia time (minutes), DBD | 700 | 520 |
| **Machine perfusion and metabolic parameters** | | |
| Lactate (mmol/L) |  |  |
| Start (0-hour) | 12.8 (9.5-16.1) | 11.3 (11.0-11.6) |
| Highest | 14.1 (9.5-20.0) | 13.5 (13.0-13.9) |
| Last (12-hours) | 0.6 (0.4-0.9) | 1.4 (1.0-1.9) |
| Sodium bicarbonate 8.4% supplementation (mL) | 15 (10-20) | 35 (30-40) |
| Total bile production (mL/hour) | 1.7 (1.7-1.7) | 0.5 (0.4-0.7) |
| Bile pH (12 hours) | 7.9 (7.9-8.0) | 7.4 (7.1-7.6) |
| Median arterial flow (mL/min) | 395 (380-410) | 259 (158-360) |
| Median portal vein flow (mL/min) | 1515 (1510-1520) | 1175 (900-1450) |
| Viability achievement | 2 (100%) | 1 (50%) |
| Urea – 12 hours (mmol/L) | 25 (23-28) | 28 (22-34) |
| Glucose – 12 hours (mmol/L) | 21 (17-26) | 28 (17-38) |
| Tissue-Triglycerides – 0-hour (mg/gr liver) | 70.1 (43.0-97.4) | 69.8 (42.7-96.5) |
| Tissue-Triglycerides – 6-hours (mg/gr liver) | 35.0 (20.1-50.7) | 65.7 (39.8-92.2) |
| Tissue-Triglycerides – 12-hours (mg/gr liver) | 43.1 (27.7-65.0) | 62.3 (37.6-87.0) |
| **Metabolism / Mobilization of fatty acids** | | |
| Total Ketone Bodies – 12-hours (mM) | 8 (3-12) | 6 (4-8) |
| ATP – 0-hours (pmole/mg liver) | 671 (227-1116) | 1485 (1270-1700) |
| ATP – 6-hours (pmole/mg liver) | 1094 (343-1845) | 485 (410-560) |
| ATP – 12-hours (pmole/mg liver) | 878 (466-1289) | 639 (596-682) |
| total-Cholesterol – 12-hours (mg/dL) | 41 (39-43) | 12 (4-19) |
| Perfusate triglycerides – 0-hours (mg/dL) | 71.3 (35.0-106.8) | 106.0 (71.1-141.6) |
| Perfusate triglycerides – 6-hours (mg/dL) | 96.4 (80.1-112.4) | 84.2 (71.9-97.0) |
| Perfusate triglycerides – 12-hours (mg/dL) | 225.4 (177.1-274.6) | 133.5 (116.0-142.7) |
| **Markers of hepatocellular injury** | | |
| ALT – 12-hours (IU/L) | 3387 (2620-4155) | 15671 (13585-17757) |
| TNFα – 12-hours (pg/mL) | 108 (85-131) | 2055 (118-3992) |
| IL1β – 12-hours (pg/mL) | 17 (12-22) | 1128 (41-2217) |
| IL10 – 12-hours (pg/mL) | 859 (275-1444) | 5210 (2482-7937) |
| 4-HNE – 6-hours (IRS points) | 1 (1-2) | 2 (2-3) |
| CD14 – 6-hours (IRS points) | 3 (3-4) | 6 (5-7) |
| CD11b – 6-hours (IRS points) | 2 (2-3) | 6 (4-9) |

**Abbreviation:** DCD- Donation after circulatory death; UK- United Kingdom; ET- Eurotransplant; ALT- Alanine aminotransferase; GGT- Gamma-glutamyltransferase; DBD- Donation after brain death; ATP- Adenosine triphosphate; TNFα- Tumour necrosis factor alpha; IL- Interleukin; 4-HNE- 4-Hydroxynonenal; IRS- Immunoreactive score; CD- Cluster of differentiation.

**2.5. Table S5: Clinical studies involving the individual components utilised in the combination of defatting agents employed in this study.**

| **Compound** | **Evidence available** |
| --- | --- |
| **Glucagon mimetic cAMP activator forskolin** | Forskolin is commercially available as a dietetic supplement. It was previously tested in two clinical series, whereby its safety to humans was shown (1,2).  *[1] Godard, MP, et al. Body Composition and Hormonal Adaptations Associated with Forskolin Consumption in Overweight and Obese Men. Obesity Research 2005, 13: 1335-1343. [2] Henderson S, et al. Effects of Coleus Forskohlii Supplementation on Body Composition and Hematological Profiles in Mildly Overweight Women. Journal of the International Society of Sports Nutrition 2005, 2(2):54-62.* |
| **Peroxisome proliferator‐activated receptors (PPAR) GW7647** | GW7647 was not clinically used thus far. Previous *in vitro* experiments have shown that GW7647 within a combination of defatting drugs is not toxic to primary human hepatocytes, human cholangiocytes, human sinusoidal endothelial cells (1). It was shown to be safe when tested *in vitro* in human skeletal muscle cells (2).  *[1] Boteon YL, et al. An effective protocol for pharmacological defatting of primary human hepatocytes which is non-toxic to cholangiocytes or intrahepatic endothelial cells. PLoS ONE 2018, 13(7): e0201419. [1] Muoio DM, et al. Peroxisome Proliferator-Activated Receptor-α Regulates Fatty Acid Utilization in Primary Human Skeletal Muscle Cells. Diabetes 2002, 51(4): 901-909.* |
| **Pregnane X receptor (PXR) ligand Hypericin** | Hypericin is commercially available in dietetic supplements. Due to an antidepressant effect, hypericin was broadly investigated in clinical studies, and even a Cochrane meta-analysis, which have shown its safety for human use (1,2,3).  *[1] Linde K, et al. St John's wort for depression: meta-analysis of randomised controlled trials. Br J Psychiatry 2005, 186:99-107. [2] Linde K, et al. St. John's wort for depression. Cochrane Database Syst Rev 2000, (2):CD000448. [3] Kasper S, et al. Efficacy of St. John's wort extract WS 5570 in acute treatment of mild depression: a reanalysis of data from controlled clinical trials. Eur Arch Psychiatry Clin Neurosci 2008, 258(1):59-63.* |
| **Constitutive androstane receptor ligand Scorparone** | Scoparone is an active component of the Artemisia capillaris, an herbal medicine which has been traditionally used in Eastern countries for many years. Clinical studies demonstrating its effectiveness are lacking, however diverse *in vitro* and animal studies support its advantages (1,2).  *[1] Lee SH, et al. Scoparone attenuates RANKL-induced osteoclastic differentiation through controlling reactive oxygen species production and scavenging. Exp Cell Res 2015, 331(2):267-77. [2] Liu X, et al. Scoparone attenuates hepatic stellate cell activation through inhibiting TGF-β/Smad signaling pathway. Biomed Pharmacother 2017, 93:57-61.* |
| **Insulin‐mimetic adipokine visfatin** | Visfatin is a human adipocytokine physiologically produced by adipose tissue. It has insulin-mimetic and glucose-lowering properties (1).  *[1] Fukuhara A, et al. Visfatin: a protein secreted by visceral fat that mimics the effects of insulin. Science 2005, 307:426–430.* |
| **Peroxisome proliferator‐activated receptors (PPAR) GW501516** | Initial clinical trials have shown the benefits of GW501516 in improving the metabolic performance of humans (1,2). However, clinical investigation was stopped due to the association of high doses of GW501516 with the development of cancer in rats and mice (3,4). Conversely, subsequent studies found that GW501516 can even inhibit the growth *in vitro* of human cancer cell lines (MCF7 – breast cancer; UACC903 – melanoma) (5). Moreover, an *in vitro* study investigating a wide range of human tumour cell lines (HT29, HCT116, LS-174T, HepG2 and HuH7) conclude that GW501516 do not potentiate tumorigenesis in humans (6).  *[1] Barish GD, et al. "PPAR delta: a dagger in the heart of the metabolic syndrome". J. Clin. Invest 2006, 116(3):590–597. [2] Uwe D, et al. "The Peroxisome Proliferator-Activated* *Receptor β/δ Agonist, GW501516, Regulates the Expression of Genes Involved in Lipid Catabolism and Energy Uncoupling in Skeletal Muscle Cells". Molecular Endocrinology 2003, 17(12):2477–2493. [3] Geiger LE, et al. PS 895 - Rat carcinogenicity study with GW501516, a PPAR delta agonist. 48th Annual Meeting of the Society of Toxicology. 2009, Baltimore: Society of Toxicology. p. 105. [4] Newsholme SJ, et al. PS 896 - Mouse carcinogenicity study with GW501516, a PPAR delta agonist (PDF). 48th Annual Meeting of the Society of Toxicology. 2009, Baltimore: Society of Toxicology. p. 105. [5] Girroir EE, et al. Peroxisome proliferator-activated receptor-beta/delta (PPARbeta/delta) ligands inhibit growth of UACC903 and MCF7 human cancer cell lines. Toxicology 2008, 243(1-2):236-43.*  *[6] Hollingshead HE, et al. Peroxisome proliferator-activated receptor-beta/delta (PPARbeta/delta) ligands do not potentiate growth of human cancer cell lines. Carcinogenesis 2007, 28(12):2641-9.* |

**2.6. Figure S1**

**Lactate clearance dynamics and perfusion parameters in the context of defatting of human livers.** Series 1: The graph shows the targets analysed for dynamic changes in the lactate metabolism over time. Series 2: for the assessment of dynamic changes in lactate concentration in the perfusate over time the area under the curve (AUC) was determined for each liver. Lactate AUC values from time 0 to 12 hours were compared with other perfusion parameters. There was a strong association between smaller values of lactate AUC with improvements in other metabolic parameters of the organ. Labels identify livers from different groups in the graph. Series 3: the time to achieve the lactate peak (in hours) was correlated with functional parameters and perfusion features. A shorter time to peak of lactate was associated with improvement in diverse parameters of metabolic function of the organ. Series 4: investigation of the correlation between time to lactate less than 2.5 mmol/L (in hours) and perfusion/ metabolic parameters. Data showed that shorter time to reach this target is associated with enhanced metabolic parameters. Pearson’s r was used to estimate the relationship between the variables.


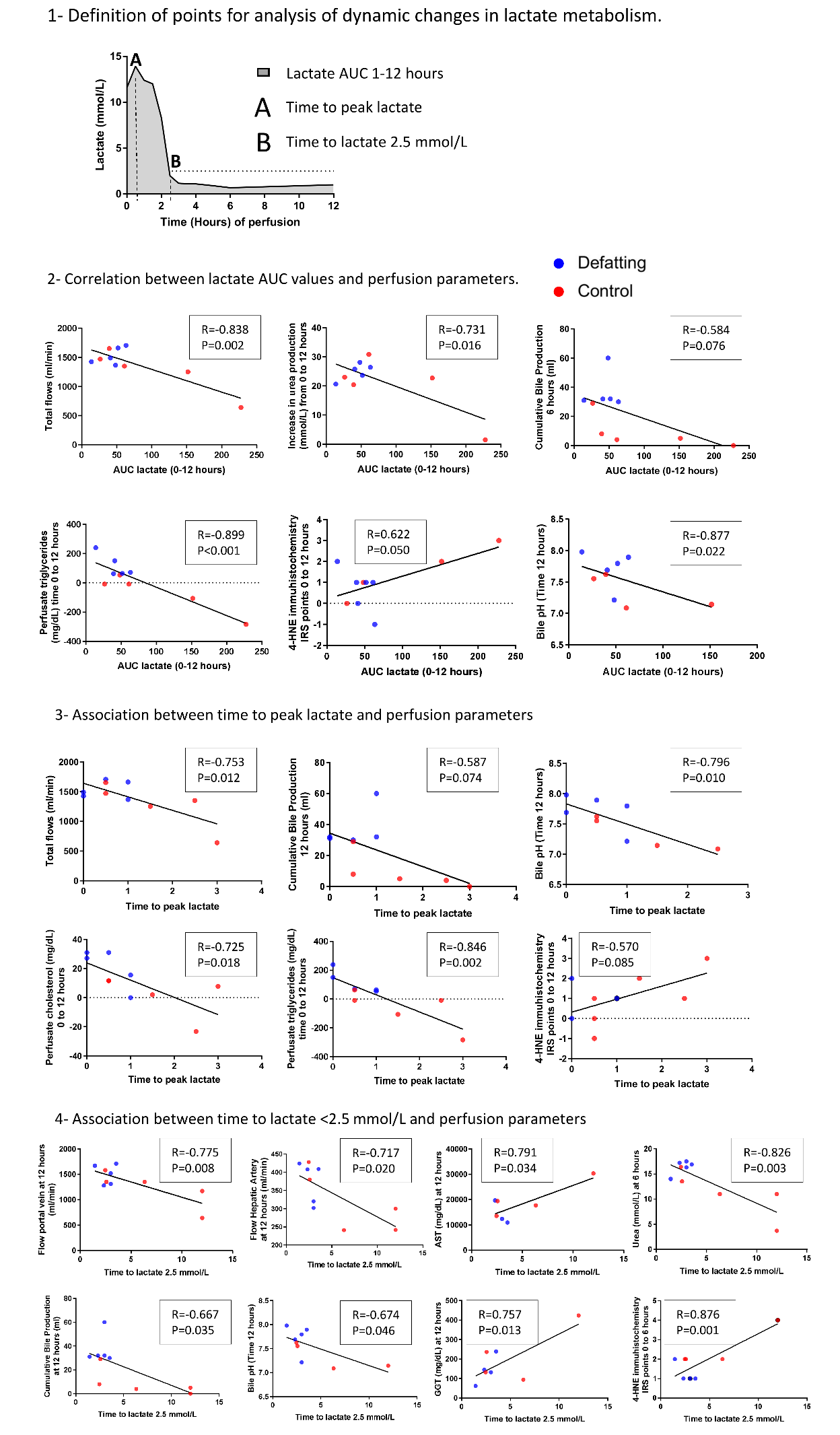


**2.7. Figure S2**

**Biochemistry analysis of the perfusate.** Panel A: Total proteins (TPROT) were measured in the perfusate and levels were comparable between groups. Panel B: Albumin presented a rising trend in the defatting group and it was stable in the control group. Panel C: Sodium levels measured in the perfusate were comparable between groups. Panel D: Potassium levels decreased quicker within 6 hours of perfusion in the defatting group and for the control levels decreased less and then were flat after initial 2-3 hours. Panel E: Perfusate pH tended to be higher in the control group.


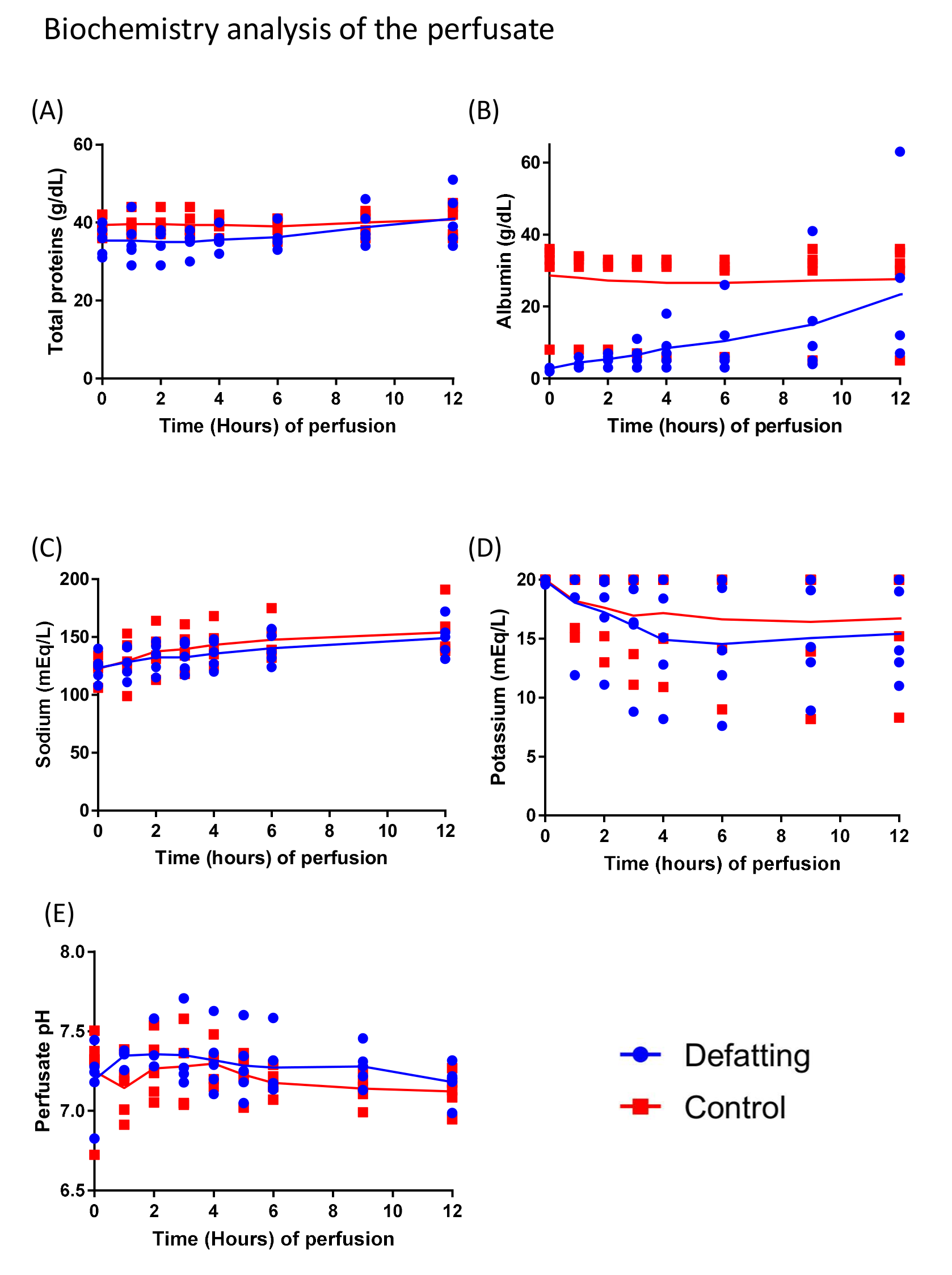

Supplement: Supplementary file 1 [file LT-25-1007-s001.docx]
